# *Rubia cordifolia* L. Dichloromethane Extract Ameliorates Contrast-Induced Acute Kidney Injury by Activating Autophagy via the LC3B/p62 Axis

**DOI:** 10.3390/molecules31020316

**Published:** 2026-01-16

**Authors:** Xiaoying Sun, Kangxu He, Guanzhong Chen, Xiaoda Yang, Xinhui Pan, Kai Liao

**Affiliations:** 1 Key Laboratory of Xinjiang Phytomedicine Resource and Utilization, Ministry of Education, Institute for Safflower Industry Research, School of Pharmacy, Shihezi University, Shihezi 832002, China; s17731511105@163.com (X.S.); hkx16699040910@163.com (K.H.); cgz970718@163.com (G.C.); 2 College of Nursing and Health, Hebei College of Science and Technology, Tangshan 063200, China; 3 Stake Key Laboratory of Natural and Biomimetic Drugs, Department of Chemical Biology, School of Pharmaceutical Sciences, Peking University, Beijing 100191, China; xyang@bjmu.edu.cn

**Keywords:** *Rubia cordifolia* L., dichloromethane extract, contrast-induced acute kidney injury, apoptosis, LC3B/p62, autophagic

## Abstract

Contrast-induced acute kidney injury (CIAKI) has emerged as the third most prevalent etiology of clinically acquired acute kidney injury, with a lack of specific preventive and therapeutic strategies. *Rubia Cordifolia* L. (madder root), a medicinal herb with a long-standing history and extensive clinical application, exhibits multiple pharmacological activities. This study aimed to clarify the renal protective effect of *Rubia cordifolia* L. dichloromethane extract (RCDE) on CIAKI modeling rats and investigate potential anti-apoptotic and autophagy-inducing effects molecular mechanisms. In this study, RCDE constituents were identified by UPLC-Q-TOF-MS. A CIAKI rat model was established to evaluate the nephroprotective effect of RCDE. The results showed that RCDE high-dose group significantly decreased serum SCr and BUN levels, attenuated renal histopathological damage, and modulated oxidative stress markers by decreasing MDA and CAT while increasing SOD, compared with the model group. It downregulated the expressions of Bcl-2, caspase-3 and p62, upregulated the expressions of Bax, Beclin1 and reduced the LC3B-II/LC3B-I ratio in renal tissues. Molecular docking indicates that anthraquinone compounds are probably the principal active constituents of RCDE. This study provides experimental evidence for the intervention efficacy of RCDE against CIAKI.

## 1. Introduction

Contrast-induced acute kidney injury (CIAKI) is defined as an acute decline in renal function following intravenous exposure to iodinated contrast media [1,2]. It is currently considered to rank third among the causes of hospital-acquired AKI [3,4]. Despite intravenous hydration being the mainstay for CIAKI prevention, its renal protective efficacy is suboptimal in high-risk populations [2,5].

Clinical studies indicated that after administration, contrast media are exclusively excreted by the kidneys of patients [6,7]. After glomerular filtration, contrast agents escape renal tubular reabsorption, resulting in a progressive increase in their tubular concentration [1,8,9]. The diverse pathological pathways are implicated in CIAKI [10]. These pathways include hypoxic injury, reactive oxygen species (ROS)-mediated oxidative stress [11], inflammatory response [12], and apoptotic processes [11,13,14]. Of these, ROS-driven oxidative stress has been identified as a primary contributor to contrast-induced cell death by activating caspase-dependent apoptosis and impairing autophagic flux [14,15,16,17].

Mitophagy plays an important role in CIAKI [18]. As a survival route, mitophagy can eliminate damaged mitochondria and reactive ROS and prevent cell death by regulating genes associated with programmed cell death [16]. Studies have shown that PINK1-PRKN mediated mitophagy can inhibit apoptosis and tissue damage [12,19], overexpression of BNIP3-induced mitophagy also exerts a protective effect in CIAKI rat model [11]. Meanwhile a variety of compounds can exert renoprotective effects by inducing mitophagy, such as paricalcitol and tetramethylpyrazine on CIAKI [5,19,20]. In addition, disrupted autophagic flux, typically characterized by abnormal accumulation of LC3B-II and p62 [21,22], impairs the clearance of damaged organelles such as dysfunctional mitochondria and cytotoxic aggregates, thereby triggering excessive apoptotic responses in renal tubular cells and further amplifying kidney damage [23]. Therefore, inducing mitophagy has emerged as a highly promising therapeutic strategy for alleviating renal injury caused by contrast agents. However, reports on relevant drug interventions for this approach are relatively scarce.

*Rubia cordifolia* L., commonly referred to as madder, generally distributed across northeast, north, and northwest China, is a medicinal plant with a rich historical background. In recent years, the pharmacological properties of *Rubia cordifolia* L. has garnered significant attention due to their diverse therapeutic potential, such as anti-inflammatory [24], antioxidant [25], mitochondrial protective [26,27], and anti-apoptotic [28,29] effects across various pathological models. Current research has demonstrated that *Rubia cordifolia* L. dichloromethane extract (RCDE) exerts the principal bioactive properties described for this plant [24]. It has exhibited promising renoprotective potential in anti-tumor [30], antioxidative [31], anti-platelet aggregation [27,32], and anti-inflammatory [25] effects in various experimental models of renal injury. Although preliminary reports have indicated therapeutic potential against CIAKI, the definitive efficacy and detailed molecular underpinnings, including specific signaling pathways, require further investigation.

This study aims to investigate the renoprotective efficacy of RCDE against CIAKI in animal models and explore the potential autophagy-inducing effects of RCDE and elucidate the underlying mechanisms, thereby providing a theoretical foundation for its future clinical translation.

## 2. Results

### 2.1. UPLC-Q-TOF-MS Analysis of Rubia cordifolia L. Dichloromethane Extract

**Figure 1:**
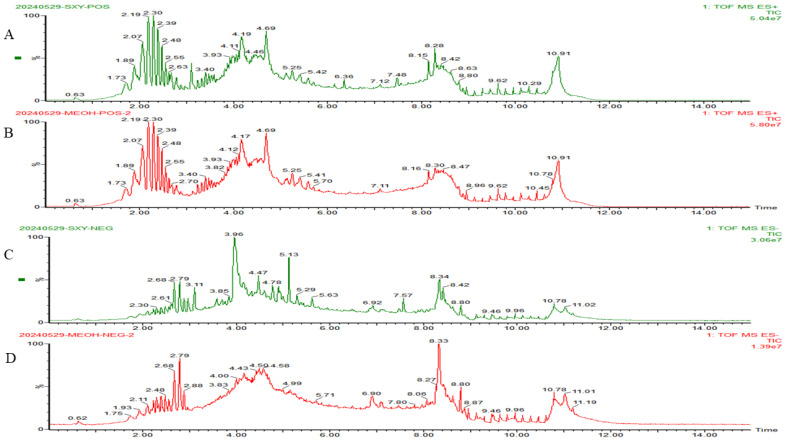
HPLC-Q-TOF-MS total ion current chromatograms of RCDE. (**A**) Total ion current chromatogram of RCDE in POS; (**B**) Total ion current chromatogram of methanol blank in positive ion mode; (**C**) Total ion current chromatogram of RCDE in NEG; (**D**) Total ion current chromatogram of methanol blank in negative ion mode.

The UPLC-Q-TOF-MS was employed to analyze the chemical constituents of RCDE and conducted a comparison with a compound database. Based on the alignment of experimental data with database references, a total of 13 major compounds in RCDE were identified, as shown in Figure 1 and Table 1 and Table 2. Specifically, 5 of these compounds are anthraquinones. These results provide essential chemical information for further investigating RCDE’s properties.

### 2.2. Effects of RCDE on Serum BUN, SCr, and Kidney/Body Weight Ratio in CIAKI Rats

**Figure 2:**
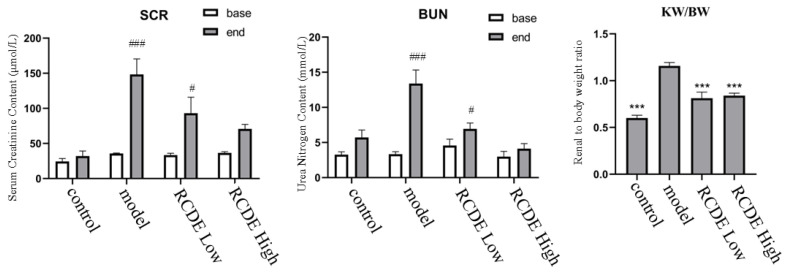
Levels of SCr and BUN in serum of CIAKI rats (*n* = 5, mean ± SD). ***: Compared with the model group, *p* < 0.001, #: Compared with the baseline (base) group, *p* < 0.05, ###: Compared with the baseline (base) group, *p* < 0.001.

To investigate the effect of RCDE on renal function in CIAKI rats, a CIAKI rat model was established. As shown in Figure 2, in the model group, the post-modeling levels of Serum Creatinine (SCr) and Blood Urea Nitrogen (BUN) were increased by 315.24% (*p* < 0.001) and 299.41% (*p* < 0.001) compared with their respective baseline values. The high-dose RCDE group showed much lower increases in SCr and BUN, which were 93.46% and 38.13%, respectively. The kidney/body weight of the model group was significantly higher than that of the control group (*p* < 0.001), while it in both RCDE groups was significantly lower than that in the model group (*p* < 0.001).

### 2.3. HE Staining

**Figure 3:**
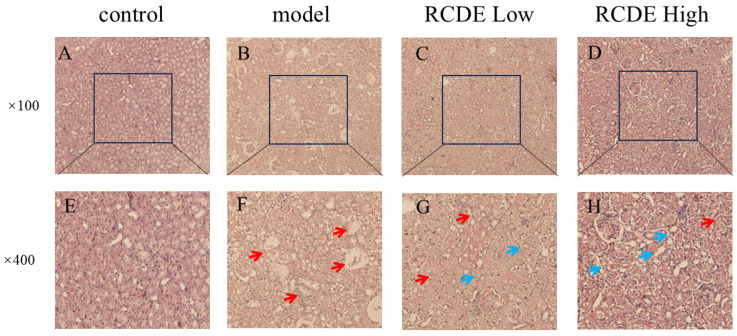
HE staining of renal tissues (*n* = 5). (**A**,**E**): Control group; (**B**,**F**): Model group; (**C**,**G**): Low-dose group; (**D**,**H**): High-dose group. The red arrows indicate the injured areas, while the blue arrows denote the recovered regions.

To observe the effects of RCDE on the kidneys of CIAKI rats, Hematoxylin and eosin (HE) staining was employed. The results (Figure 3) indicated that renal tubules in the control group displayed intact tissue structure and regularly arranged cells under microscopic observation at 400× magnification. The model group exhibited severe renal tubular obstruction and granular degeneration, along with massive inflammatory cell infiltration and cellular vacuolation. In both the high-dose and low-dose RCDE groups, renal tubules showed partial recovery, with the disappearance of tubular obstruction. In addition, the high-dose group showed reduced inflammatory cell infiltration in renal tubules, while infiltration persisted in the low-dose group but was less severe than in the model group.

### 2.4. RCDE Attenuates Renal Oxidative Damage in Contrast-Induced Acute Kidney Injury

**Figure 4:**
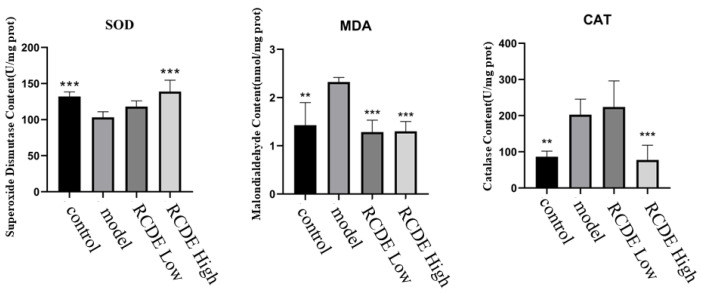
The effect of RCDE on the content of SOD, MDA and the activity of CAT in renal tissue of CIAKI rats (*n* = 5, mean ± SD). **: Compared with the model group, *p* < 0.01; ***: Compared with the model group, *p* < 0.001.

Given that oxidative stress injury frequently occurs in CIAKI, the oxidative stress status of the kidneys was detected. As shown in Figure 4, compared with the control group, the activity of SOD in the model group decreased significantly, while the contents of MDA and CAT increased significantly. Pre-intervention with different doses of high-does RCDE, compared with the model group, exhibited a significant decrease in MDA content and CAT activity as well as a significant increase in SOD activity in renal tissues (all *p* < 0.001).

### 2.5. TUNEL

**Figure 5:**
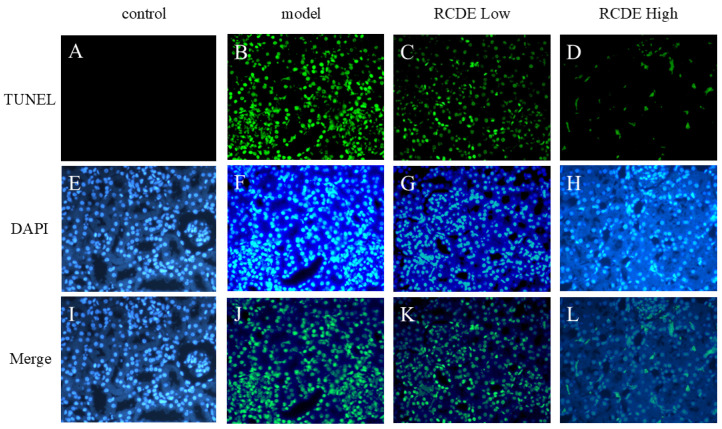
Comparative analysis of TUNEL staining across different experimental groups (*n* = 5, 200×). The figure presents fluorescence microscopy images of (**A**–**D**) TUNEL staining, (**E**–**H**) DAPI staining, and (**I**–**L**) merged images combining both channels. The columns represent four distinct experimental conditions: Control (**A**,**E**,**I**), Model (**B**,**F**,**J**), RCDE Low-dose treatment (**C**,**G**,**K**), and RACE high-dose treatment (**D**,**H**,**L**).

As depicted in Figure 5, in comparison to the control group, the CIAKI model group exhibited a significant elevation in the quantity of Terminal deoxynucleotidyl transferase dUTP Nick End Labeling (TUNEL)-positive cells within renal tissues. The administration of high-dose RCDE led to a substantial reduction in model-induced TUNEL-positive cell. This finding suggests that high-dose RCDE exerts a potent inhibitory effect on renal cell apoptosis in CIAKI rats.

### 2.6. Western Blot

**Figure 6:**
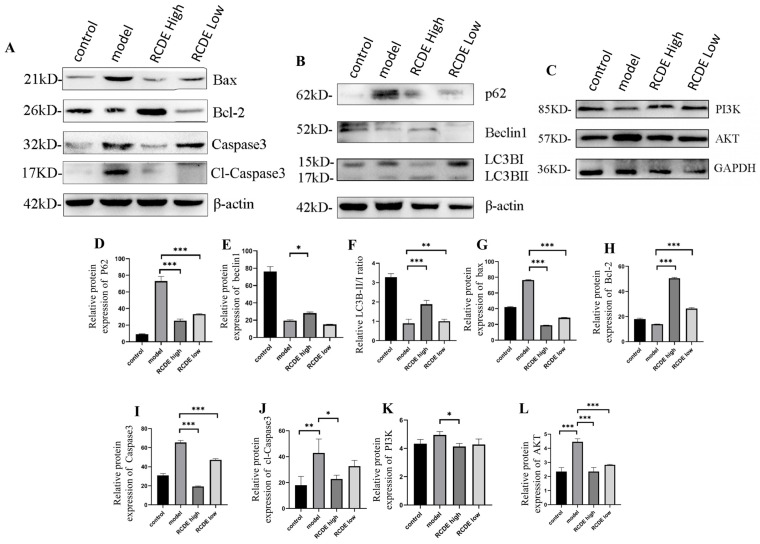
RCDE Regulates Apoptosis and Autophagy-related Protein Expression in Renal Tissues of CIAKI Rats (*n* = 3). (**A**) Representative WB bands of apoptosis-related proteins (Bax, Bcl-2, Caspase3); (**B**) autophagy-related proteins (p62, Beclin1, LC3B-I/II) with β-actin as the loading control; and (**C**) PI3K, AKT with GAPDH as the loading control (**D**–**L**) Quantitative analysis of relative protein expression levels (normalized to β-actin and GAPDH). The data are presented as mean ± SD from three independent biological replicates. * *p* < 0.05, ** *p* < 0.01, *** *p* < 0.001 vs. model group.

To further elucidate the protective mechanism of RCDE against CIAKI, the expression levels of apoptosis-related key proteins (Bcl-2, Bax, caspase-3, cl-caspase-3) and autophagy-related proteins (LC3B, p62, beclin1, PI3K, AKT) were quantitatively detected using western blot (WB). β-actin and GAPDH served as an internal reference for protein quantification normalization. As illustrated in Figure 6, compared with the CIAKI model group, the high-dose RCDE group exhibited regulatory effects on the expression of target proteins in renal tissues. Regarding apoptosis-associated proteins, an up-regulation of Bcl-2 (*p* < 0.001) and a down-regulation of protein Bax (*p* < 0.001), caspase-3 (*p* < 0.001) and cl-caspase-3 (*p* < 0.05) were observed in the high-dose RCDE group, which collectively contribute to inhibiting renal cell apoptosis (Figure 6B). For autophagy-related proteins, the high-dose RCDE group can significantly upregulate PI3K protein (Figure 6C, *p* < 0.05) expression while concurrently downregulating AKT (Figure 6C, *p* < 0.001), thereby promoting autophagic flux via modulation of the PI3K/AKT signaling pathway. The autophagic substrate p62 showed a significant decrease in the high-dose RCDE group (Figure 6A, *p* < 0.001), suggesting enhanced autophagic degradation activity. The autophagy initiating protein Beclin1 was also reduced (*p* < 0.01). In addition, the LC3B-II/I ratio was significantly upregulated in the high-dose RCDE group (*p* < 0.001).

### 2.7. Observation of Mitochondria via TEM

**Figure 7:**
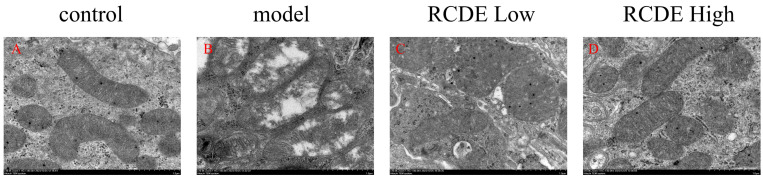
Effects of RCDE on mitochondrial autophagy (*n* = 3, 10,000×). (**A**): Control group; (**B**): Model group; (**C**): Low-dose group; (**D**): High-dose group.

Transmission electron microscopy (TEM) was utilized to examine the ultrastructure of rat renal tissues. In the control group, mitochondria presented with well-preserved structures, featuring smooth outer membranes and an abundance of cristae formed on the inner membranes. In the model group, however, a state of severe mitophagy was noted. Some mitochondria had ruptured, and there was a marked reduction in the number of inner cristae. Regarding the RCDE low-dose group, a partial restoration of mitochondrial structure was observable. As for the RCDE high-dose group, only a minority of mitochondria still exhibited damage (Figure 7).

### 2.8. Molecular Docking Analysis

**Figure 8:**
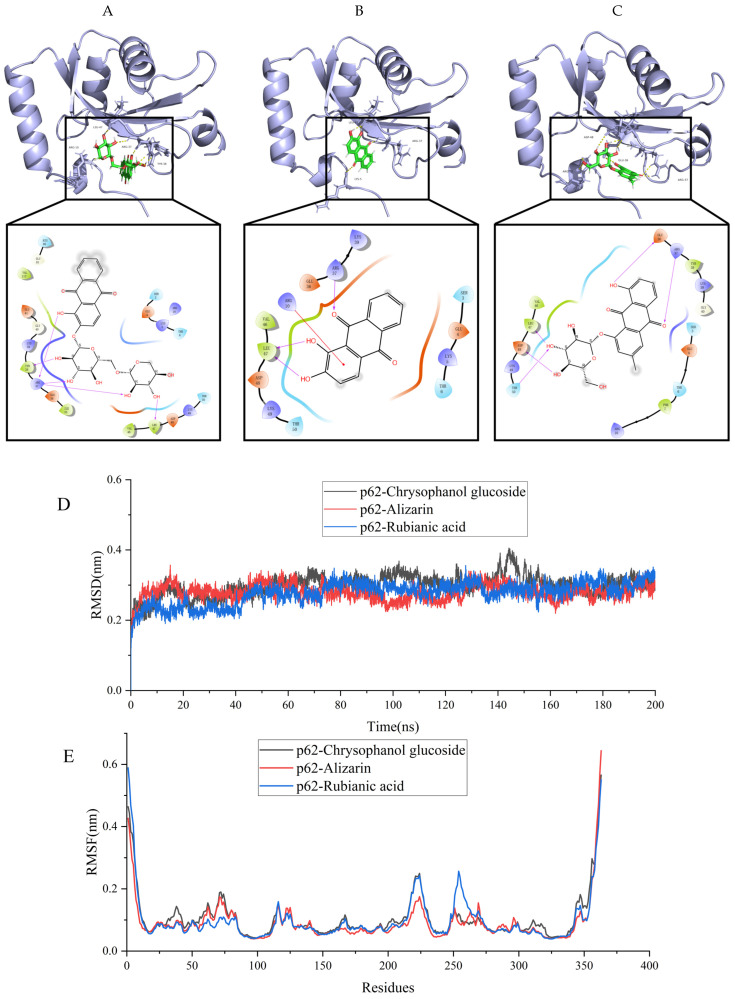
Visualization of molecular docking between p62 and the main chemical components of RCDE. (**A**) Rubianic acid; (**B**) Alizarin; (**C**) Chrysophanol glucoside; (**D**) Time-dependent RMSD of modeled to p62 with Rubianic acid, Alizarin and Chrysophanol glucoside in 200 ns of MDs; (**E**) RMSF analysis of MDs for protein–ligand complexes.

The molecular docking analysis revealed that p62 exhibited the most favorable binding interactions with rubianic acid, alizarin, and chrysophanol glucoside (Figure 8). The binding mode for the protein-ligand complexes was depicted in Figure 8. All protein-ligand complexes exhibited binding energies below −6 kcal/mol, suggesting a strong likelihood of binding interactions. And rubianic acid, alizarin, and chrysophanol glucoside all belong to the anthraquinone class of compounds. Therefore, these results collectively suggest that anthraquinone may mediate autophagy effects via the inhibition of p62 activity.

The stability of protein binding to small molecule ligands can be shown by the molecular dynamics simulation (MDs) process. Thus, 200 ns MDs was used to assess the binding stability of p62 protein to Chrysophanol glucoside, Alizarinn, and Rubianic acid. The fluctuations of RMSD values are shown in Figure 8D. All compounds reached a stable bingding stat with the protein complex system., and the RMSD values fluctuated steadily between 0.2 and 0.4 nm, and the complex p62-Rubianic acid exhibit a better dynamic steady (0.27 ± 0.03) compared with the p62-Chrysophanol glucoside (0.30 ± 0.03) and p62-Alizarinn (0.28 ± 0.02). Furthermore, during the whole simulation process, The RMSD of the p62-Rubianic acid complex system had the lowest fluctuation, indicating that the ligand-protein binding ability was stronger, which is consistent with the docking results.

In the p62 complexes (Figure 8E), the root means square fluctuation (RMSF) curves for the p62-Chrysophanol glucoside, p62-Alizarinn, and p62-Rubianic acid complexes show considerable overlap, with both complexes exhibiting minimal fluctuations in amino acid residues between positions 150 and 200. The RMSF values for both complexes remain below 0.6 nm, indicating that the binding of the compounds does not induce significant fluctuations in the amino acid residues of p62. This suggests a high degree of stability for both complexes.

## 3. Discussion

Currently, the clinical incidence rate of CIAKI is approximately 3–15%, and it can exceed 50% in high-risk patients [33]. Moreover, CIAKI significantly prolongs patients’ hospital stays, elevates the risk of complications, and even increases long-term mortality [10]. Therefore, the prevention of CIAKI is of great clinical urgency. Previous studies have demonstrated that the primary pathogenesis of CIAKI lies in its ability to promote oxidative stress, apoptosis, and autophagy [15,18,34]. *Rubia cordifolia* L. has been reported to possess antioxidant and anti-apoptotic properties [25,35]. The effects of its active constituents from dichloromethane extracts on CIAKI were systematically investigated in this study [36].

CIAKI is clinically characterized by a 25% or greater increase in SCr levels from baseline within 2 to 5 days following contrast agent administration [37]. Consistent with other CIAKI rat models, our study demonstrated that a 48 h water deprivation, combined with furosemide (FM) administration and contrast injection in rats, led to marked reductions in renal function, deterioration of renal tissue histopathology, oxidative stress, and renal tissue cell apoptosis [31,38]. Pre-administration of oral RCDE, at both high and low doses, effectively ameliorated the aforementioned pathological progression, exhibiting significant preventive effects.

Among the 13 compounds analyzed via UPLC-Q-TOF-MS, Chrysophanol exerts anti-renal fibrosis activity by inhibiting renal interstitial fibroblast activation and extracellular matrix deposition, thereby delaying chronic kidney disease progression [39]. In other disease contexts, Alizarin can reduce protein oxidative damage as a free radical scavenger against hydroperoxyl radicals [36]. Additionally, chrysophanol shows broad pharmacological activities such as antioxidant effects [40], anti-inflammatory actions [41]. These substances represent the potential material basis for RCDE in mitigating CIAKI. Meanwhile, molecular docking analysis further revealed the interaction patterns and binding affinities between these compounds and P62, providing important clues for understanding their potential biological activities at the molecular level. Despite the current absence of direct reports regarding the relevant activities of Rubianic acid, this result provided a novel intervention strategy for CIAKI.

Activation of the PI3K/AKT signaling pathway has been shown to induce autophagic flux [42], thereby ameliorating CIAKI through enhanced cellular stress tolerance and mitochondrial quality control [43]. In the context of autophagy, LC3B and p62 assume indispensable roles [21,44]. LC3B is widely acknowledged as a marker protein that is intimately involved in the formation and expansion of autophagosomes [17,45]. This process is of paramount importance as it enables the encapsulation of cellular components designated for degradation [46]. Conversely, p62 functions as a cargo receptor, which specifically binds to ubiquitinated proteins and facilitates their delivery to autophagosomes for subsequent degradation [47]. Dysregulation of either LC3B or p62 can disrupt the normal autophagic flux, leading to the accumulation of damaged cellular components and potentially triggering cellular dysfunction [48,49]. Inducing autophagy can clear damaged mitochondria, reduce ROS levels, and exert an apoptosis-ameliorating effect in CIAKI [50].

In this study, after establishing a CIAKI rat model and administering RCDE intervention, the ratio of LC3BII to LC3BI in renal tissues significantly increased. Given that LC3BII is a hallmark protein of the autophagosome membrane, an elevated ratio of LC3BII to LC3BI generally indicates an increased formation of autophagosomes [45]. Under the condition of normal autophagolysosomal degradation function, this suggests that autophagic flux is restored and its activity is enhanced in the CIAKI rat model [51]. The strengthened autophagic process can efficiently clear the ROS generated by iohexol-induced oxidative stress. Meanwhile, it can also specifically recognize and eliminate damaged mitochondria through the autophagic mechanism [46]. The present research results are consistent with the conclusions reported in previous studies [15,52]. It not only further clarifies the molecular mechanism underlying the action pathway of RCDE in the onset and progression of CIAKI but also provides a solid theoretical basis for the potential application of RCDE as an intervention means in the clinical treatment of CIAKI, while providing substantial experimental support.

## 4. Materials and Methods

### 4.1. Animals

Twenty male Sprague Dawley (SD) rats, weighing (180 ± 20 g), were purchased from Henan Scrubs Biotechnology Co., Ltd. (Zhengzhou, China), with production license number: SCXK (Henan) 2020-0005. The rats were housed in Shihezi University’s animal experimental feeding center with a 12 h light/12 h dark cycle. Humidity was maintained at around 55%. They were fed standard rodent chow from Beijing Huafukang Biotechnology Co., Ltd. (Beijing, China) and had ad libitum access to purified water. After the experiment, the rats were euthanized via anesthesia with a 20% urethane solution. The animal experimental protocol has been approved by the Experimental Animal Ethics Committee of the First Affiliated Hospital of Shihezi University Medical College [Approval No.: A2024-006-01; Approval date: 9 January 2024]. All rats were fed adaptively for one week before the experiment.

### 4.2. Chemicals and Regents

*Rubia cordifolia* L., purchased from Wuhan Shijian Bencao Culture Co., Ltd. (Wuhan, China, Batch No.: 2022121068), was authenticated by Professor Yun Zhu from the College of Pharmacy, Shihezi University, and confirmed to be the root of *Rubia cordifolia* L. Iohexol injection, used as the contrast agent, was purchased from Yangtze River Pharmaceutical Group Co., Ltd. (Taizhou, China, Batch No.: 20123061), containing 35 g iodine per 100 mL. FM was obtained from Shanxi Zhaoyi Biotechnology Co., Ltd. (Xinzhou, China, Batch No.: 230302). Commercial assay kits for malondialdehyde (MDA), superoxide dismutase (SOD), creatinine (CRE), and blood urea nitrogen (BUN) determination were purchased from Nanjing Jiancheng Bioengineering Institute (Nanjing, China). The batch numbers are as follows: MDA-20231012, SOD-2023111, CRE-20231122, BUN-20231110. Radio Immunoprecipitation Assay (RIPA) lysis buffer was purchased from Beijing Solarbio Science & Technology Co., Ltd. (Beijing, China). The batch number is as follows: R0010. Bicinchoninic Acid (BCA) protein concentration determination was purchased from Beijing Solarbio Science & Technology Co., Ltd. The batch number is as follows: PC0020. Enhanced Chemiluminescence (ECL) detection kit was purchased from Affinity (San Francisco, CA, USA). The batch number is as follows: KF8003. Goat anti-mouse immunoglobulin G (IgG) secondary antibody (Batch No.: ZB-2305) and β-actin primary antibody (Batch No.: TA-09) were supplied by Beijing Zhongshan Jinqiao Biotechnology Co., Ltd. (Beijing, China). Primary antibodies against BAX (Batch No.: CY5059), BCL-2 (Batch No.: CY5032), and Caspase 3 (Batch No.: CY5051) were obtained from Shanghai Boway Biotechnology Co., Ltd. (Shanghai, China). The p62 primary antibody (Batch No.: GB11513-100), LC3B primary antibody (Batch No.: GB113801-100), and Beclin1 primary antibody (Batch No.: GB11513-100) were all purchased from Servicebio (Wuhan, China). The AKT (Batch No.: CY5561) and PI3K (Batch No.: CY5355) antibodies were purchased from Shanghai Abwiz Biotechnology Co., Ltd. (Shanghai, China).

### 4.3. Extraction of Dichloromethane-Soluble Compounds from Rubia cordifolia L. (Madder Root)

One hundred grams of powdered roots and rhizomes of *Rubia cordifolia* L. were weighed and immersed in a 90% ethanol solution. Reflux extraction was performed at 90 °C for 30 min, and this procedure was repeated three times. The supernatants were then combined and concentrated via reduced-pressure distillation to yield a madder ethanol extract paste. The paste was dissolved in water and subsequently extracted three times each with petroleum ether and dichloromethane (DCM) separately. Following reduced-pressure distillation, the DCM layer containing the target dichloromethane-soluble compound was collected. After removing residual moisture by drying over anhydrous sodium sulfate, the DCM layer underwent another round of reduced-pressure concentration, ultimately resulting in the acquisition of the dichloromethane-soluble extract. This final extract was stored at 4 °C for subsequent experimental use.

### 4.4. UPLC-Q-TOF-MS Analysis

The chromatography analysis was performed on an ACQUITY UPLC^®®^ BEH C18 (Waters Corporation, Milford, MA, USA; 1.7 μm, 2.1 mm × 100 mm), the flow rate was set at 0.4 mL/min, the injection volume was 2.0 μL, the column temperature was set at 45 °C. The mobile phase gradient elution conditions are shown in Table 3. The mass spectrometric (MS) was performed with electrospray ionization (ESI), and the ion modes were positive and negative. interaction mode, ion source temperature 110 °C, the dissolvent gas was nitrogen at a flow rate of 650 L/h and a temperature of 450 °C. Capillary voltage was set at 3.0 kV in positive ion mode and 2.5 kV in negative ion mode, with a cone voltage of 50 V. The mass scan range was 100–1500 Da. The collision energy was maintained at 20–50 eV for both positive and negative ion scanning. Accurate mass calibration was performed using a leucine enkephalin (LE) solution (200 pg/mL), with *m*/*z* 556.2771 for positive mode and *m*/*z* 554.2615 for negative mode. Data acquisition was carried out without correction. The Ultra Performance Liquid Chromatography-Quadrupole-Time of Flight Mass Spectrometry (UPLC-Q-TOF-MS; Waters Corporation, Milford, USA) system was operated using MassLynx 4.1 software. The acquired MS data were imported into UNIFI and matched against a natural product database for peak identification and annotation.

### 4.5. Establishment and Grouping of Rat Models of CIAKI

**Figure 9:**
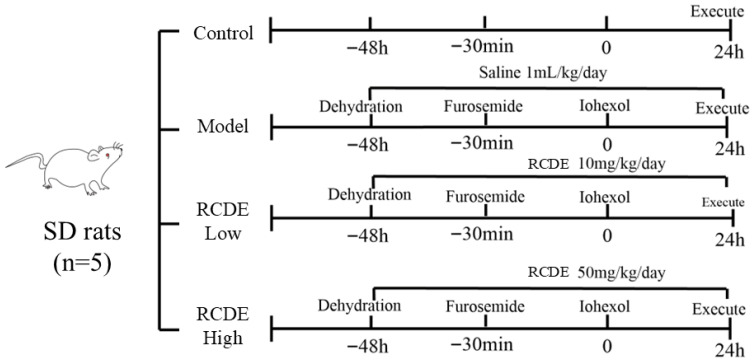
Schematic diagram of CIAKI rat modeling.

The protocol for establishing the CIAKI rat model and administering RCDE intervention is illustrated in Figure 9. Briefly, rats were subjected to water deprivation (with free access to food) for 48 h to induce mild hypovolemia, a predisposing factor for CIAKI. Subsequently, all rats (except those in the control group) received an intramuscular injection of FM at a dose of 10 mL·kg^−1^ to further promote diuresis and exacerbate renal stress. Thirty minutes after FM injection, iohexol was administered via tail vein injection at a dose of 15 mL·kg^−1^ to induce CIAKI.

A total of 20 rats were randomly divided into 4 groups (*n* = 5 per group) using a random number table: Control group: Rats received no treatment (no water deprivation, no drug/injection administration) throughout the experimental period. Model group: Rats underwent the full CIAKI modeling procedure (water deprivation + FM + iohexol) and were given an oral gavage of double-distilled water (1 mL·kg^−1^·day^−1^) for 3 consecutive days before modeling (instead of RCDE). Low-dose RCDE group: Rats received oral gavage of RCDE at a dose of 10 mg/kg/day for 3 consecutive days prior to CIAKI modeling (the same modeling procedure as the model group). High-dose RCDE group: Rats received oral gavage of RCDE at a dose of 50 mg/kg/day for 3 consecutive days prior to CIAKI modeling (the same modeling procedure as the model group).

Twenty-four hours after iohexol administration, all rats were anesthetized with 10% chloral hydrate (3 mL/kg, intraperitoneal injection) for sample collection. Blood samples were harvested via abdominal aorta puncture, and renal tissues were immediately excised, rinsed with pre-cooled physiological saline to remove residual blood, and processed for subsequent experiments (histological analysis, biochemical detection, and Western blot assay).

### 4.6. Determination of Kidney-to-Body Weight Ratio

After the experimental rats were sacrificed, bilateral renal tissues were immediately harvested. The collected renal tissues were rinsed gently with pre-cooled physiological saline to remove surface blood stains, then blotted dry with sterile filter paper to eliminate excess moisture. The wet weight of the cleaned renal tissues was accurately measured using an electronic analytical balance (Mettler-Toledo Instruments Co., Ltd., Shanghai, China). All measurements were performed in duplicate to ensure data reproducibility, and the average value was used for subsequent statistical analysis.

### 4.7. Determination of Renal Function Indicators

Blood samples (0.6 mL each) were collected from the orbital venous plexus of rats at two time points: 30 min before CIAKI modeling (baseline) and 24 h after iohexol administration (post-modeling). During blood collection, rats were gently restrained. Under mild isoflurane anesthesia to minimize stress-induced fluctuations in renal function parameters, the orbital venous plexus was punctured using a sterile, heparinized capillary tube to prevent coagulation.

The collected blood samples were immediately centrifuged at 3500× *g* for 15 min at 4 °C to separate the serum. The supernatant (serum) was carefully aspirated with a pipette and transferred to sterile microcentrifuge tubes, which were stored at −80 °C until analysis to prevent analyte degradation.

Serum levels of SCr and BUN, two classic biomarkers of renal glomerular filtration function, were measured using commercial assay kits (Nanjing Jiancheng Bioengineering Institute, Nanjing, China; Batch No. 20231122 for SCr, Batch No. 20231110 for BUN) according to the manufacturer’s standard protocols. Absorbance readings were obtained using a Varioskan Flash automatic microplate reader (Thermo Fisher Scientific, Waltham, MA, USA; Model: VARIOSKAN FLASH), and the concentrations of SCr and BUN were calculated based on standard curves generated in parallel with each assay run. All determinations were performed in triplicate to ensure analytical accuracy.

### 4.8. HE Staining of Renal Tissues

Freshly excised rat renal tissues were immediately fixed in 4% paraformaldehyde (*w*/*v*, dissolved in phosphate-buffered saline, PBS, pH 7.4) at room temperature for 24 h to preserve tissue morphology. After fixation, the samples were dehydrated through a graded series of ethanol (70%, 80%, 90%, 95%, and absolute ethanol, 15 min each), cleared in xylene (twice, 15 min each), and embedded in paraffin wax (melting point: 56–58 °C) to prepare paraffin blocks.

Serial sections (4 μm thick) were cut from the paraffin blocks using a microtome (Leica Biosystems, Wetzlar, Germany) and mounted on polylysine-coated glass slides to prevent detachment during staining. The sections were then deparaffinized with xylene (twice, 10 min each) and rehydrated through a reversed ethanol gradient (absolute ethanol, 95%, 90%, 80%, 70%, 5 min each) before being rinsed with distilled water for 5 min. For HE staining, the rehydrated sections were stained with hematoxylin solution (Sigma-Aldrich, St. Louis, MO, USA) for 5 min, differentiated with 1% hydrochloric acid in ethanol (*v*/*v*) for 30 s, and blued in tap water for 10 min. Subsequently, the sections were counterstained with eosin solution (Sigma-Aldrich) for 3 min, followed by dehydration through graded ethanol (70%, 80%, 90%, 95%, absolute ethanol, 3 min each) and clearing in xylene (twice, 5 min each). Finally, the stained sections were mounted with neutral balsam and coverslipped.

### 4.9. TUNEL Staining

Renal paraffin sections (4 μm thick) were deparaffinized, rehydrated, and washed with PBS. Sections were permeabilized with 1× proteinase K at 37 °C for 20 min, then rinsed with PBS.

TUNEL reaction mixture was added, and sections were incubated at 37 °C for 60 min in the dark. After PBS washing, sections were counterstained with DAPI (1 μg/mL) for 10 min, then mounted with anti-fluorescence quenching medium.

Images were captured under a fluorescence microscope (Carl Zeiss (Oberkochen, Germany), Imager.M2) at 200× and 400× magnifications. TUNEL-positive (green) and DAPI-stained (blue) cells were counted via ImageJ (https://imagej.net/ij/ (accessed on 3 October 2025)). The apoptotic index was calculated as (TUNEL-positive cells/total nuclei) × 100%, with analyses performed by two blinded researchers.

### 4.10. Detection of Oxidative Stress Levels in Renal Tissues

Renal tissue samples (approximately 100 mg) were homogenized in pre-cooled physiological saline (1:9, *w*/*v*) using a tissue homogenizer (Beyotime Biotechnology, Shanghai, China) on ice to prepare 10% tissue homogenates. The homogenates were centrifuged at 3500× *g* for 15 min at 4 °C, and the supernatants were collected for subsequent assays.

The levels of MDA and the activities of SOD and CAT were determined using commercial assay kits strictly following the manufacturer’s protocols.

Absorbance values were measured with a Varioskan Flash automatic microplate reader (Thermo Fisher Scientific Inc., Waltham, MA, USA). MDA content was expressed as nmol/mg protein, while SOD and CAT activities were presented as U/mg protein, with protein concentrations in tissue supernatants quantified using the BCA protein assay kit (Beijing Solarbio Science & Technology Co., Ltd., Beijing, China) to standardize the results. All detections were performed in triplicate.

### 4.11. Western Blot Analysis

Renal tissue proteins were extracted on ice using RIPA lysis buffer supplemented with 0.1% PMSF to prevent protein degradation. Protein concentration in each sample was determined with a BCA protein assay kit to ensure equal loading. Equal amounts of protein (30 μg per lane) were separated by 10–12% SDS-PAGE and then electrotransferred onto PVDF membranes using a wet transfer apparatus (Beijing Solarbio Science & Technology Co., Ltd., Beijing, China). After transfer, the membranes were blocked with 5% non-fat milk (dissolved in Tris-buffered saline with Tween 20, TBST) at room temperature for 2 h to block non-specific binding sites. Subsequently, the membranes were incubated overnight at 4 °C with primary antibodies: anti-BAX (1:1000), anti-BCL-2 (1:1000), anti-caspase 3 (1:1000), anti-p62 (1:500), anti-LC3B (1:500), anti-β-actin (1:1500), anti-Beclin1 (1:1000), anti-PI3K (1:1000), and anti-AKT (1:1000). The next day, the membranes were washed three times with TBST (10 min each) and then incubated with HRP-conjugated goat anti-mouse IgG secondary antibody (1:5000) at room temperature for 1.5 h. After three additional TBST washes, protein bands were visualized using an ECL detection kit and imaged with a gel imaging system (Shanghai Tianneng Technology Co., Ltd., Shanghai, China). The band densities were quantified using ImageJ software, and the values were normalized to that of β-actin (or GAPDH) for statistical analysis.

### 4.12. Molecular Docking

The Chem3D 17.0 software was employed to minimize the energy of the active compounds. The crystallographic structures of the proteins relevant to the target genes were obtained from the Protein Data Bank (PDB) database (https://www.rcsb.org). Molecular docking simulations were performed with the Maestro 12.8 software (Grid-based Ligand Docking with Energetics) to determine the binding affinities and poses of the active compounds with the receptor proteins. LigPrep preprocessed ligands to establish physiological charge states and minimum-energy conformers. Concurrently, Maestro’s Protein Preparation Wizard refined downloaded protein structures. Finally, the Receptor Grid Generation module defined the docking pocket grid. The centroid of the cocrystal ligand within the crystal structure was designated as the center of the active site, and a grid file with dimensions of 20 × 20 × 20 Å was generated and computed. Ultimately, molecular docking was conducted on the prepared files using the SP-precision algorithm of the Glide docking module. The binding interactions between the active compounds and the target proteins were evaluated based on their binding affinities. Scores for binding energy below −6 kcal/mol signify strong, favorable binding interactions. Visualization of the docking outcomes was conducted with PyMOL software (https://pymol.org/ (accessed on 9 October 2025)).

### 4.13. Molecular Dynamics Simulations

Small molecule-target protein complexes with the lowest absolute binding free energy in molecular docking were selected for molecular dynamics simulations using the Gromacs2023 software package for the Frog Hopping Newton Integral Algorithm, which is used for equilibrium kinetic integration. During the simulation, Amber99SB force field parameters were used to generate the protein topology, and the small molecule ligands were first calculated using Multiwfn for RESP2(0.5) (http://sobereva.com/476 (accessed on 11 October 2025)) charge, followed by Sobtop (http://sobereva.com/soft/Sobtop (accessed on 11 October 2025)) software to generate the GAFF force field topology. The TIP3P water model was used to add solvent to the protein-ligand system. Then, cubic boxes were built and supplemented with Na^+^/Cl^−^ to equilibrate the system. The energy of the complex system was optimized using the 50,000-step most rapid descent method. After the system energy optimization was completed, the system temperature was steadily increased from 0 K to 300 K at a fixed volume and constant heating rate. 100 ps NVT (isothermal and isovolumetric) system simulations were performed to uniformly distribute the solvent molecules in the solvent at a system sustaining temperature of 300 K. The system was then optimized by the most rapid descent method at 50,000 steps to equilibrate the system. Then, a 100 ps NPT (isothermal and isobaric) system simulation was performed for the composite system at one atmosphere. The simulation was performed with a time step of 2 fs and the simulation trajectory was saved every 100 ps. Finally, a 200 ns molecular dynamics simulation was performed and the trajectories were used to analyze the root mean square deviation (RMSD).

### 4.14. Statistical Analysis

All experimental data were analyzed and visualized with GraphPad Prism 8.2 software (GraphPad Software, San Diego, CA, USA), and presented as mean ± SD. For multi-group comparisons (e.g., renal index, SCr, BUN, or protein expression levels among control, model, low and high-dose RCDE groups), one-way ANOVA with Tukey’s post hoc test was used. For data with two independent variables (e.g., effects of treatment group and time point on renal function indicators at baseline and 24 h post-modeling), two-way ANOVA with Bonferroni’s post hoc test was applied. *p*-value < 0.05 was considered statistically significant. All experiments were repeated at least three times for data reliability.

## 5. Conclusions

In CIAKI rats, RCDE intervention significantly restored renal function, attenuated pathological damage, and reduced cellular apoptosis, demonstrating its therapeutic potential. Importantly, this protection was mediated via activation of the LC3B/P62-dependent autophagy pathway. To explore its active components, 13 major compounds were identified in RCDE using UPLC-Q-TOF-MS analysis, and their binding affinities for the autophagy-related protein P62 were evaluated via molecular docking simulations. Among these, rubianic acid, alizarin, and chrysophanol glucoside exhibited high-affinity interactions with P62, suggesting their direct involvement in autophagy regulation.

Key limitations include a small per-group sample size (*n* = 5), which may compromise statistical robustness, and reliance on in silico docking without functional validation. Consistent with our findings, recent studies [11,53] have demonstrated that targeting autophagy pathways significantly ameliorates CIAKI progression, further supporting RCDE’s mechanism of action. The findings suggest that the components of *Rubia cordifolia* L. could serve as candidate agents for the intervention of CIAKI. Furthermore, subsequent research will be conducted to further investigate the intervention effects of its individual compounds on CIAKI.

## Figures and Tables

**Table 1:** Chemical Constituents Identified in RCAE via UPLC-Q-TOF-MS Under Positive Ion Modes.

Peal	RT/min	Identified Anthraquinone Compound	Molecular Formula	[M+H]^+^	[M+H]^+^ Calculated	Error (ppm)
1	2.63	d-Corydaline	C_22_H_27_NO_4_	369.1940	369.1933	−1.68
2	6.36	Xantholide A	C_15_H_18_O_2_	230.1307	230.1307	0.00
3	7.48	Chrysophanol glucoside ^a^	C_21_H_20_O_9_	416.1107	416.1077	−6.73
4	8.63	Cimicifugadine ^a^	C_35_H_51_NO_8_	613.3614	613.3622	1.19
5	8.80	Meranzin hydrate	C_15_H_18_O_5_	278.1154	278.1178	8.70

^a^ Measured mass [M+Na]^+^.

**Table 2:** Chemical Constituents Identified in RCAE via UPLC-Q-TOF-MS Under Negative Ion Modes.

Peal	RT/min	Identified Anthraquinone Compound	Molecular Formula	[M+H]^−^	[M+H]^−^ Calculated	Error (ppm)
1	2.61	Psoralen ^a^	C_11_H_6_O_3_	186.0317	186.0327	4.50
2	3.11	Isaindigodione ^a^	C_20_H_18_N_2_O_4_	350.1267	350.1291	6.06
3	3.96	Alizarin	C_14_H_8_O_4_	240.0423	240.0437	6.04
4	5.13	Rubianic acid	C_25_H_26_O_13_	534.1373	534.1324	−9.02
5	5.29	Catenarin	C_15_H_10_O_6_	286.0477	286.0491	4.70
6	5.63	1-Hydroxy-2-hydromethylanthraquinone	C_15_H_10_O_4_	254.0579	254.0591	4.56
7	7.57	3β-Acetoxy-16α-hydroxylanosta-8,24-dien-21-oic acid	C_32_H_50_O_5_	514.3658	514.3669	2.19
8	8.42	Ganolactone ^a^	C_27_H_36_O_6_	456.2512	456.2486	−5.07

^a^ Measured mass [M+HCOO]^−^.

**Table 3:** Mobile phase gradient elution conditions for samples.

Time (min)	A (%)	B (%)	Curve
0	5	95	-
10	95	5	6
12	95	5	6
13	5	95	6
15	5	95	6

## Data Availability

The data presented in this study are available on request from the corresponding authors.

## Abbreviations

The following abbreviations are used in this manuscript:

CIAKI	Contrast-induced acute kidney injury
RCDE	*Rubia cordifolia* L. dichloromethane extract
UPLC-Q-TOF-MS	Ultra-high performance liquid chromatography quadrupole time-of-flight tandem mass
SD	Sprague–Dawley
MDA	Malondialdehyde
CAT	Catalase
SOD	Superoxide dismutase
BCA	Bicinchoninic acid
RIPA	Radio-Immunoprecipitation Assay Lysis Buffer
SCr	Serum Creatinine
BUN	Blood Urea Nitrogen
HE	Hematoxylin and Eosin
TUNEL	TdT-mediated dUTP Nick-End Labeling
